# Race and statistics in facial recognition: Producing types, physical attributes, and genealogies

**DOI:** 10.1177/03063127221127666

**Published:** 2022-10-27

**Authors:** Abigail Nieves Delgado

**Affiliations:** Utrecht University, The Netherlands

**Keywords:** principal component analysis (PCA), statistics, facial recognition, race, ontology of the normal

## Abstract

Principal component analysis (PCA) is a common statistical procedure. In forensics, it is used in facial recognition technologies and composite sketching systems. PCA is especially helpful in contexts with high facial diversity, which is often translated as racial diversity. In these settings, researchers use PCA to define a ‘normal face’ and organize the rest of the available facial diversity based on their resemblance to or difference from that norm. In this way, the use of PCA introduces an ‘ontology of the normal’ in which expectations about how a normal face should look are corroborated by statistical calculations of normality. I argue that the use of PCA can lead to a statistical reification of racial stereotypes that informs recognition practices. I discuss current and historical cases in which PCA is used: one of face perception theorization (‘face space theory’) and two of technology development (the ‘eigenfaces’ facial recognition algorithm and the ‘EvoFIT’ composite sketching system). In each, PCA aligns facial normality with racial expectations, and instrumentalizes race in specific ways: as a type, physical attribute, or genealogy. This analysis of PCA does two things. First, it opens the black box of facial recognition to uncover how stereotypes and intuitions about normality become part of theories and technologies of facial recognition. Second, it explains why racial categorizations remain central in contemporary identification technologies and other forensic practices.

Facial recognition systems are part of a set of procedures and technologies (composite sketching, DNA identification and phenotyping, biometric identification) used in current forensic sciences to identify a person. These technologies and their underlying statistical methods order data and determine the nature of the information gathered, its relevance, and the ways it should be interpreted. These ordering processes have important societal effects, as these technologies are crucial for predicting and profiling human groups and individuals, following socially relevant categories, such as race, sex, class, and age (Eubanks, 2018).

Works in history of science and science and technology studies have studied the objectivity and accuracy of these technologies and identification practices. In particular, they focus on body measurements and criminalization (Caplan, 2001; Cole, 2002), photography (Tagg, 1993), DNA identification and phenotyping (Hopman, 2021; Lynch et al., 2008; M’charek, 2000, 2008), and the effects of these technologies on group belonging (Sekula, 1986).

Of major relevance in forensic identification is the subject of race. In particular, M’charek (2000, 2008) has shown that forensic identification depends on the production of populations of reference, a procedure that often involves racialization processes. Her work in forensics shows that race does not lie on the surface of bodies but is produced through identification procedures. In different forensic practices race is enacted in at least three ways: as biological characteristics of the body, as patterns of ‘absent presence’ in forensic analyses and finally as processes of stereotyping or phenotypic othering (M’charek, 2013; M’charek et al., 2020).

A central target of such racialization practices in identification is the human face. Current facial recognition technologies determine identity by analyzing the face in terms of visual patterns, which are detected by algorithms. Computer vision algorithms learn ‘what to see’ based on certain databases used during their training (e.g., Jaton, 2017). In this process, the (often opaque) composition of databases and the complexity of the machine learning procedures involved may obscure what happens to data, and thus to human faces.

Despite the claims of neutrality made for facial recognition processes, facial recognition technologies are often not objective and can potentiate discriminatory behaviors and biases. There is growing concern about the effects of biases (Leslie, 2020; Singer & Metz, 2019), profiling applications (Crockford, 2020; Ruppert, 2011), lost privacy, and policing technologies (Sedenberg & Chuang, 2017). The daily use of allegedly neutral technologies may exacerbate and normalize discriminatory practices. It is thus important to uncover the kinds of ordering and classificatory work these technologies perform when they recognize our faces, and the statistical processes underlying such classificatory practices. Statistics and their classificatory powers are ‘powerful technologies’ embedded in complex infrastructures (Bowker & Star, 2000, p. 319), acting on data without being detected. It is thus crucial to open the black box of facial recognition to reveal what categories, statistical processes and assumptions are shaping how our faces are recognized.

This article explores how the statistics underlying facial recognition shape and organize facial data. I aim to show how race is produced by invisible statistical procedures at work in facial recognition theories and technologies. I focus on the statistical method of principal component analysis (PCA), which has been highly influential in the development of theories and technologies of facial recognition and identification (e.g., Claes et al., 2014; Shuang, 2014). The analysis starts from the general assumption that technologies are not simple value-neutral means of obtaining particular goals, but are in fact ‘actors’ that shape our reality (Miller, 2021) and enable possibilities and practices (Suchman, 1994). Thus, this research endorses a view of technologies and methods as performative and ‘actively engaged in the constitution of the reality’ that they describe (Callon, 2006).

I explore the use of PCA in facial recognition by means of three case studies: the ‘face space’ theory (Valentine, 1991), the ‘eigenfaces’ facial recognition algorithm (Turk & Pentland, 1991), and EvoFIT, an automatized composite sketching system; (Frowd et al., 2004). In these three cases, recognition is modeled by creating a ‘face space’, a three-dimensional space by means of which faces are separated according to how they look. This organization, which I call an ‘ontology of the normal’, labels and classifies different faces as normal or abnormal. Central to this categorization is the assumption that diversity in physical appearance can be translated into racial difference. In each of the three cases discussed, the process of recognition instrumentalizes different views of race: as type, as a physical attribute, and as genealogy.

These three enactments of race are reminiscent of the history of racial science, in which typologies, body descriptions, and lineages have been constructed as evidence of racial difference (Teslow, 2014; Wade, 2019). However, in contrast to past efforts (involving portraits, measurements, and averages) in facial recognition, these racialization processes leave no material trace and are usually invisible. The three enactments of race explored here exemplify how facial recognition is utilized to draw and legitimize connections between statistically described normality and phenotypically perceived normality. Consequently, the groups created statistically by PCA are considered to match groups considered to be racially different.

This study is based on an analysis of published documents, as well as empirical material gathered through semi-structured interviews with facial recognition experts in the fields of computer vision and experimental psychology. The interviews were approximately one hour long and were conducted in person or online. Dr. Lloyd, Dr. Allison, Dr. Roberton, and Dr. Ricks (pseudonyms) are researchers whose work is central to the development and/or use of the theories presented here. The selection of the cases is based not only on their relevance to the history of their respective fields, but also to current practice and developments in identification and facial recognition. In what follows, I first introduce how facial forensic information is gathered in databases, the role PCA plays in this context, and how it has shaped the development of automatized facial recognition and identification technologies. I then analyze the way PCA organizes data in three case studies (face space theory, eigenfaces and EvoFIT), and how in each of these cases a different (but related) version of race is enacted.

## Statistics and databases in identification practices

Forensic information is nowadays organized in large databases, such as the Combined DNA Index System (CODIS) in the United States and the European Criminal Records Information System (ECRIS). To make it manageable, it is necessary to reduce such large amounts of data. Early 20th century statistics, as well as current neural network algorithms, such as those employed by Facebook and Google, are examples of data reduction technologies. Central to data reduction strategies are statistical procedures: calculations that organize data but, at the same time, invent, construct, and provide scientific facts (Desrosières, 1998, p. 3). Statistics not only makes data manageable but, at the same time, produces an ontology that determines the very nature of the information gathered, its relevance and the ways it should be interpreted (Kruse, 2013). Applied to human groups, statistics produces the categories that are used to describe them, and, as a consequence, the populations to be described (see Serre & Pääbo, 2004) – a phenomenon Hacking (2007) has famously termed ‘making up people’. Thus, statistics is performative in the sense that it enacts and describe realities (Law, 2009).

The emergence and the overarching adoption of statistics to regulate society has been studied in relation to, among other things, the organization of populations of modern states (Desrosières, 1998; Espeland & Stevens, 2008), as part of a trend toward quantification and objectivity in science and society (Porter, 1995) and connected to British biometrics and eugenics in the early 20th century (MacKenzie, 1981). These discussions on the effects of quantification and governance in society are currently taking a new turn with the introduction of algorithmic technologies and data collection practices in big data settings, which should enable the predicting and profiling of human groups and individuals (Ruppert, 2011).

In forensic sciences and biometric technologies, data collection practices and underlying statistical procedures produce groupings of humans following relevant social categories, such as race, sex, and age (e.g., Tokola et al., 2015). In these grouping processes, statistics organize human bodies according to specific phenotypic characteristics, which should provide knowledge about those groups and the individuals included in them. Faces are statistically grouped and analyzed using categories such as race and sex, based on facial features considered to be relevant (shapes, shadows, and distances of points and patterns). Such approaches to categorizing the human face are not new. Over the centuries, practices like physiognomy and physical anthropology have given the face multiple meanings, which have accumulated on its surface: including, among others, race, disease, intelligence, and crime (M’charek & Schramm, 2020; Nieves Delgado, 2020a; Percival, 1999).

In contrast to those previous technologies and practices, current facial recognition technologies draw on computer vision algorithms that analyze and identify faces in terms of visual patterns. Through machine learning these algorithms learn what to see and how to distinguish between faces in a database. In this training process the composition of the database, or so-called ‘ground truth’, is of great relevance, as its content defines what the algorithms can do (Jaton, 2017). Dr. Lloyd, a pioneer in the field of facial recognition, has emphasized that ‘facial recognition is blind without a database’. What makes automated facial recognition novel is the current computational capacity to collect, save, share, and re-use huge databases of information. For Dr. Lloyd, the combination of good cameras and social networks drives technology development through daily, continuous, and voluntary contributions by users to the creation of vast pools of photographs. This combination makes facial recognition ubiquitous and more powerful than ever before. This means that if facial recognition relies on racial categories to work – and this paper aims to show that it does – then racialization processes and their consequences for society are also intensified to a level never seen before.

Databases are key in this development. Scholars in science and technology studies have studied databases in relation to their curation (Leonelli, 2016; Pinel et al., 2020), datafication practices (Hoeyer et al., 2017, 2019) and their effects in scientific research and knowledge production (Mayernik, 2019), as well as in regard to privacy (Lupton, 2020). I contribute to this discussion by looking at the statistical tools used to organize data in databases, the ontologies they create, and their influence on how we use this data. This especially concerns PCA, a central statistical tool in the cases explored here. Drawing on a performative understanding of technologies, I seek to uncover how the production of facial normality mobilizes and enacts specific conceptions of race.

## PCA: Producing normal faces from data

There are various scientific strategies used to determine how best to describe a group of individuals. In the study of human variation, charts and measurements represented a way to obtain descriptions of the characteristics of a population, which were usually conceptualized as racial differences (Dias, 2010; Morris-Reich, 2016; Teslow, 2014). The British biometric school contributed to this effort by introducing multiple statistical methods to the study of biology and anthropology (MacKenzie, 1981). The main advocates of this view, Francis Galton (1857–1936), Walter F.R. Weldon (1860–1906) and Karl Pearson (1857–1936), developed important mathematical techniques for analyzing empirical data, and set the basis for a mathematical view of nature, including the human body.

**Figure 1.**
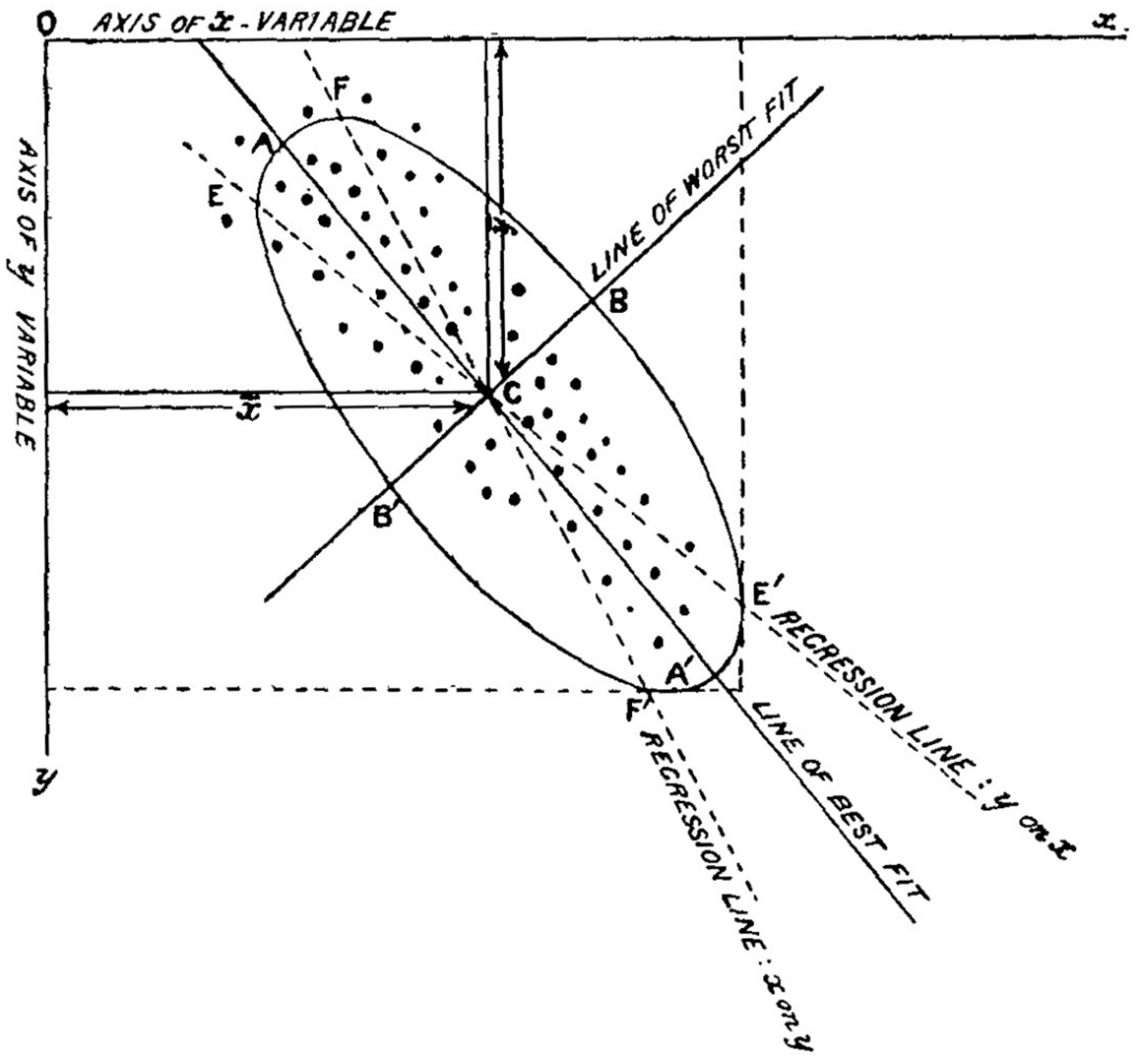
Depiction of PCA as the ‘line of best fit’. This method reduces higher-dimensional sets of data into lower-dimensional sets of relevant characteristics. Here, two variables, *x* and *y*, are singled out and represented as axes of a two-dimensional plane. The ‘line of best fit’ in the image is the principal component, which carries the most information about the data set. For details, see Pearson (1901, p. 566).

Among the statistical tools in Pearson’s legacy is PCA. Pearson introduced PCA in his paper ‘On lines and planes of closest fit to systems of points in space’, published in 1901. In this publication, he explained that in observational sciences such as physics and biology ‘it is desirable to represent a system of points in plane, three, or higher dimensioned space by the “best fitting” straight line or plane’ (Pearson, 1901, p. 559). PCA draws a line that crosses a set of observations where the relevant characteristics of a group vary the most, drawing a ‘line of best fit’ (see Figure 1). It can iteratively reduce higher-dimensional data sets to lower-dimensional sets. For example, in Figure 1, only two dimensions (i.e. the *x*- and *y*-variables) are depicted. In today’s data sciences, PCA is a central information reduction method taught in textbooks (Kong et al., 2017; Malik & Tuckfield, 2019). Among its many uses, in forensics, PCA has been applied to produced portraits from genetic information (Claes et al., 2014; for discussion see M’charek & van Oorschot, 2019) and in facial recognition, new algorithms based on PCA appear regularly (e.g., Erwin et al., 2019; Javed, 2020).

PCA works on sets of data that are given in terms of characteristics that are common to all objects within a set but that vary between objects (Abdi & Williams, 2010). A researcher measures faces, say, in length and their width, distance between eyes, size of the mouth, and distance from the mouth to the nose, to mention some possible measuring points. If the observational dataset is too large and the researcher is unable to select which of these measurements are more informative to grasp the relevant characteristics of the set, then she can apply PCA. PCA will determine which characteristics are the most relevant to describe the group of faces. These are then selected as the principal components of the group.

The calculated principal components play simultaneously a descriptive and a prescriptive role. In fact, this tension underlies the history of probability and statistics (Desrosières, 1998; Hacking, 1971). The descriptive role of statistics concerns registering the frequency of specific phenomena found in nature. In the case above, it represents what the researcher knows about the group of faces based on the available sample and the measurements taken. But it says nothing about the possible incompleteness of the sample or possible measurement errors. The second role concerns what a researcher knows, and offers guidelines for actions based on predictions made from data. It is prescriptive because it establishes how things *ought* to be based on what *is*.

The organization of the points on the plane (the faces) represents in PCA both a *description of the frequency* with which different types of faces appear and *what is expected* to be found based on the given sample. At the very center of the PCA plane we find the *statistically* normal face, the face with the most frequent characteristics in a group of faces and the one expected with highest probability (from a specific group of faces). This normal face is expected to overlap with the *experienced* normal face (the type of face we see more often and which, for that reason, looks more ordinary or typical to us). The normal face at the center of PCA works as an axis that organizes the rest of the faces according to degrees of similarity to it. In this way, PCA works by creating an *ontology of the normal*, organizing faces in relation to their similarity to a central norm.

In forensic practice, the identification of normal faces has specific challenges. It is argued that people can remember and distinguish between faces with distinctive qualities, such as a big nose or a very wide forehead, but they remember normal faces less well (Valentine & Bruce, 1986). In these challenging cases, researchers need to generate databases containing more detail (i.e., the database contains more information to describe noses, eyes, mouths, etc. of each face). To do that, they produce homogeneous databases with faces that resemble each other. The next sections examine three strategies for producing such homogeneous databases and how, through these procedures, researchers re-enact different versions of race. As we will see, the organization of facial difference through PCA fosters thinking about this difference in terms of types, physical attributes, and lineages of races.

## Face space theory: Facial recognition experiments and race

In the field of experimental psychology, face recognition and face perception are important research topics, especially for forensic purposes (Davies et al., 1981; Rivolta et al., 2018). Central in this context is the ‘face space’ theory (Valentine, 1991), which is a model for understanding how facial recognition works in humans. In 1991, numerous experiments in face recognition had shown that an individual recognizes different types of human faces more or less quickly depending on certain characteristics of these faces, such as ‘typicality’ (Kleisner et al., 2019), the position or orientation of the faces in a picture (upright vs. upside down), and race belonging (see Davies et al., 1981). In other words, these studies asked whether normal-looking faces, in contrast to distinctive ones, are recognized equally well. Dr. Allison explained the face space theory in an interview:

[H]ow do you identify easily whether people look similar or completely different from each other ?… You can think of them [the faces] as the similar ones are stored in a different location from the non-similar ones. This helps you to identify those people who are similar looking. … [A]s we develop, … every face that we encounter is stored and incorporated into a common space that has an average in the middle, an average of all the faces that you have seen is in the middle of your individual face space.

**Figure 2.**
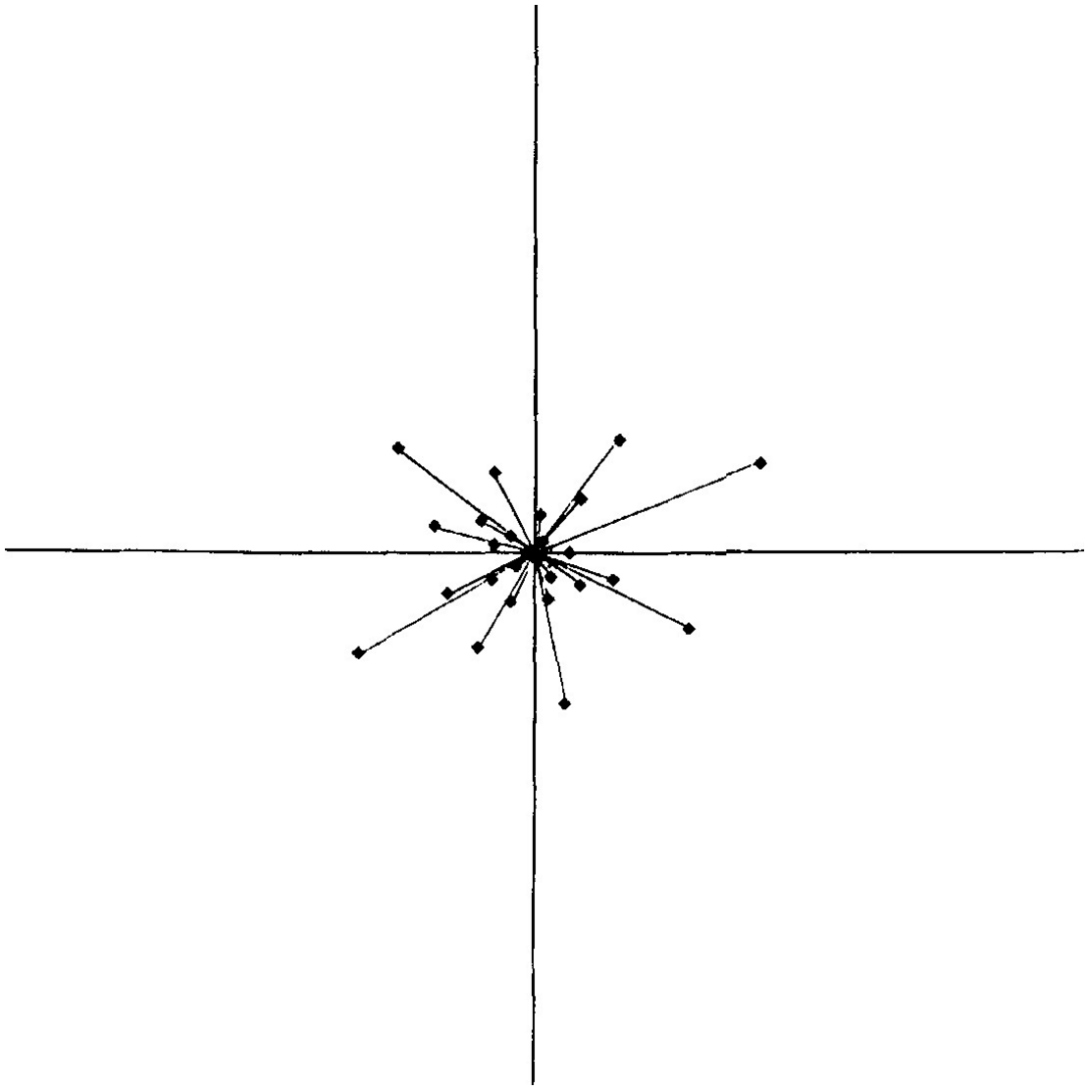
Depiction of the ‘face space’ according to the face space theory. At the center of the space we find an abstract ‘norm’ or prototype face. The rest of the faces are organized in relation to this central norm (Valentine, 1991, p. 168).

The face space theory consists of a multidimensional space (imagined as a three-dimensional Cartesian plane) where faces seen during our lifetime are saved and organized. The organization of the faces in this space is given by their characteristics (shape and size of the mouth, eye color, etc.) and resemblance to each other, where typical faces are located at the center and distinctive ones are located at the edges (see Figure 2 for a two-dimensional representation of the face space).

**Figure 3.**
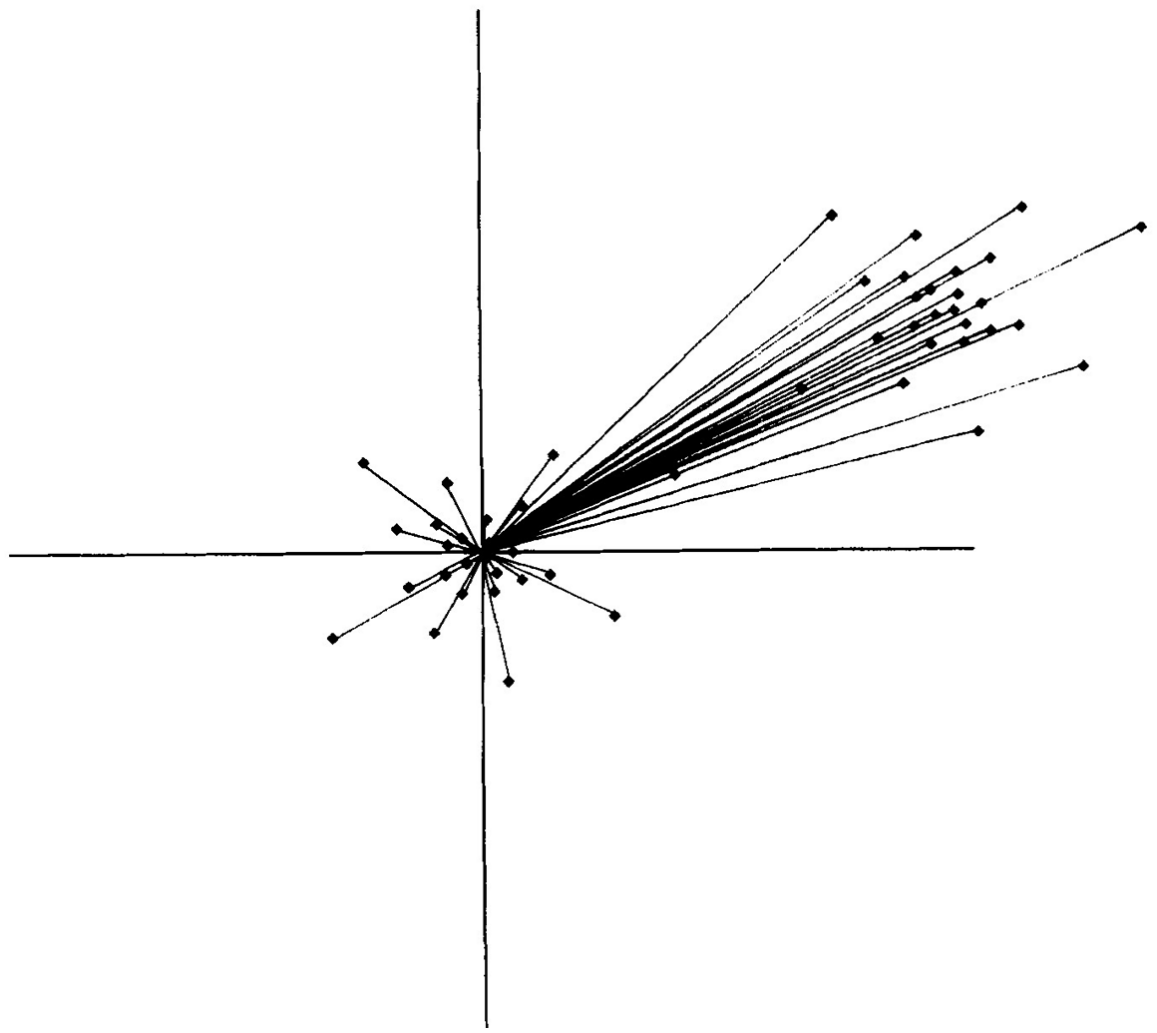
Norm-based model and own race effect (ORE). The idea behind ORE predicts that an observer will recognize the faces of those of her own race better than the faces of those of a different race. The dots located on the right side, far away from the center, indicate race differences (Valentine, 1991, p. 192).

In this arrangement, frequently observed faces occupy the center of the space, where we find a prototype face that is an average of all the faces found in the face space; the process is reminiscent of Galton’s (1879) composite portraits. This prototype organizes the face space according to the degree of resemblance between itself and the rest. Dr. Allison offers the following explanation regarding the organization of the face space: ‘There is a dimension [in the face space] for the distance between the eyes for example, so the average would be in the middle and people who have eyes very outside or very close eyes they will be at the extremes of this space’. Let’s say that in Figure 3 the face with the average distance between the eyes is located in the middle. This normal face is abstracted from all the faces seen during a person’s lifetime and the eye dimensions observed across those faces. Accordingly, people with an average distance between the eyes would be at the center and ‘people with eyes very outside’ (in Dr. Allison’s words) would be represented through the projecting lines reaching from the center to the edge of the space.

The power of the face space theory lies in its ability to explain a basic intuition we have about how we recognize people and are recognized by people. For instance, when walking down the street we may think someone has a very strange face or a very beautiful face. According to the face space approach, we judge faces in this way because we have an implicit idea of a ‘normal face’ that acts as a reference point for such judgments, based on our experience. Importantly, our ideas of normality, strangeness and beauty (to mention a few) change from individual to individual and depend on the place in which each person has lived, as we will see below.

### Race as type

Crucially, the face space theory emphasizes the geographical and biographical situatedness of individuals to explain their varying facial recognition capacity. It suggests that when a person travels or moves to a different country, she is exposed to a different set of faces. It is assumed that these faces will look different and will be considered distinctive. They are organized in the face space, but they are projected away from the center – as shown in Figure 3 (Valentine et al., 2016). This means that we develop a face space based on our life experience and the type of faces we see. However, it is not only a matter of the types of faces we see around us: our own face also matters. Dr. Allison gives an example to explain this: ‘A Caucasian baby adopted in Africa will become an expert in African face recognition but also [an expert on] Caucasian [faces] just based on her own face, even if there is few [Caucasian] people around’. This assumes that there is a connection between geographic region and the facial types to be found in a given place (Nash, 2013). In other words, it is expected that people from a place will look alike, and in expected ways. This expectation of phenotypic resemblance is not new: it has been used within physical anthropology for the production of racial typologies (Broca, 1865; see Teslow, 2014) and in population genetics for the production of populations of reference (see Fujimura & Rajagopalan, 2011; M’charek, 2005; Nash, 2013). By assuming that the organization of the personal face space depends on our geographical and biographical trajectories, as well as our own face, the face space theory incorporates racial types and stereotypes in face recognition. It does so by assuming an expected implicit difference between ‘Caucasian’ and ‘African’ faces. Thus, it instrumentalizes a typological understanding of race to account for facial recognition.

The example of the baby also helps us to describe what is known as the ‘own race effect’ (ORE, see Figure 3). Due to the ORE, faces that are considered to belong to a ‘race’ are recognized faster by an observer who is also categorized as belonging to that same race. In contrast, the recognition of faces from other races is slower (Feingold, 1914). In other words, the ORE affirms that an individual is better at recognizing people of her own ‘race’ than others. Researchers have developed experiments to prove the ORE.

In experimental settings, facial homogeneity and difference is achieved following certain assumptions about the racially coded facial differences to be found in populations. Researchers select observers from (what they consider to be) one population, ethnicity, or race, who then try to recognize faces from individuals (considered to be) from the same group and from a different one. For instance, researchers tend to invite ‘White’ participants (in some studies named Caucasian), who typically are requested to recognize faces labeled as ‘White’ and ‘Black’ (Valentine & Bruce, 1986; Wan et al., 2017). Similar experimental designs ask Chinese participants to recognize Caucasian and Asian faces (He et al., 2010) and Black South African and Chinese participants to recognize each other’s faces (Zhou et al., 2015). In such studies, the process of ascribing participants and photographs to racial categories is not problematized. On the contrary, difference in racial terms is taken as a matter of fact. In these experiments each individual and the set of faces observed as stimuli are taken as representatives of types of faces associated with racial, ethnic or national groups. Based on these assumptions our recognition capacity has been theorized as follows. According to Dr. Ricks, a psychology professor who has worked extensively in facial recognition, observers pay attention to different facial traits depending on facial race type. It is said, for instance, that Black people, in contrast to White people, look more to the mouths of other Black people to identify them, as it is said that the appearance of Black people’s mouths varies a lot. Thus, the explanation is that our way of looking at others would be equivalent to PCA’s way of extracting principal components from them. Accordingly, our expertise in recognizing our own race comes from the principal components we have learned to extract. Thus, the interpretation of the face space through PCA would be an adequate representation of how our perception works. Leaving aside questions about the veracity of this theory, the example given by Dr. Ricks points to the normative role of Whiteness in facial recognition, in contrast to the alterity of Blackness. This enactment of race is further explored in the next case.

## Eigenfaces: Automated facial recognition

The modeling of the face space through PCA is central not only for experimental psychology but also for the development of face recognition tools in forensics (Claes et al., 2014; Frowd et al., 2019; O’Toole et al., 2018). One such tool is ‘eigenfaces’, a highly influential facial recognition system which operates under similar assumptions of racial difference and normality, enacting a different version of race.

Eigenfaces produces a ‘face space’ by analyzing a given database of standardized faces through PCA (Turk & Pentland, 1991; Valentine et al., 2016: 2010). In short, it extracts the characteristics that are common to a group of faces. The best-known proposal on the use of eigenfaces appeared in 1991, published by Mathew A. Turk and Alex ‘Sandy’ Pentland, two computer scientists at MIT (Turk & Pentland, 1991). They drew inspiration from the Karhunen Loève procedure introduced by Kirby and Sirovich one year before – the Karhunen Loève procedure, involving a computational version of PCA, overlaps images to extract the minimal number of features that describe that set of images (Kirby & Sirovich, 1990). It is a computational version of PCA. According to Dr. Roberton, a computer scientist who has worked in the field since the 1990s, ‘Eigenfaces for recognition’ by Turk and Pentland (1991) is one of the most cited papers in history, with 18,000 (and counting) citations. ‘It is a very basic idea and a very simple mathematical procedure’, he explains, ‘and this is the reason of its great success’. The eigenfaces approach was one of the five finalists in the facial recognition contest FERET (1993–1996) organized by the Department of Defense of the United States to evaluate the state of the art in the field and to develop facial recognition for security purposes. In this regard, right from the beginning, eigenfaces was developed specifically for surveillance and policing.

**Figure 4.**
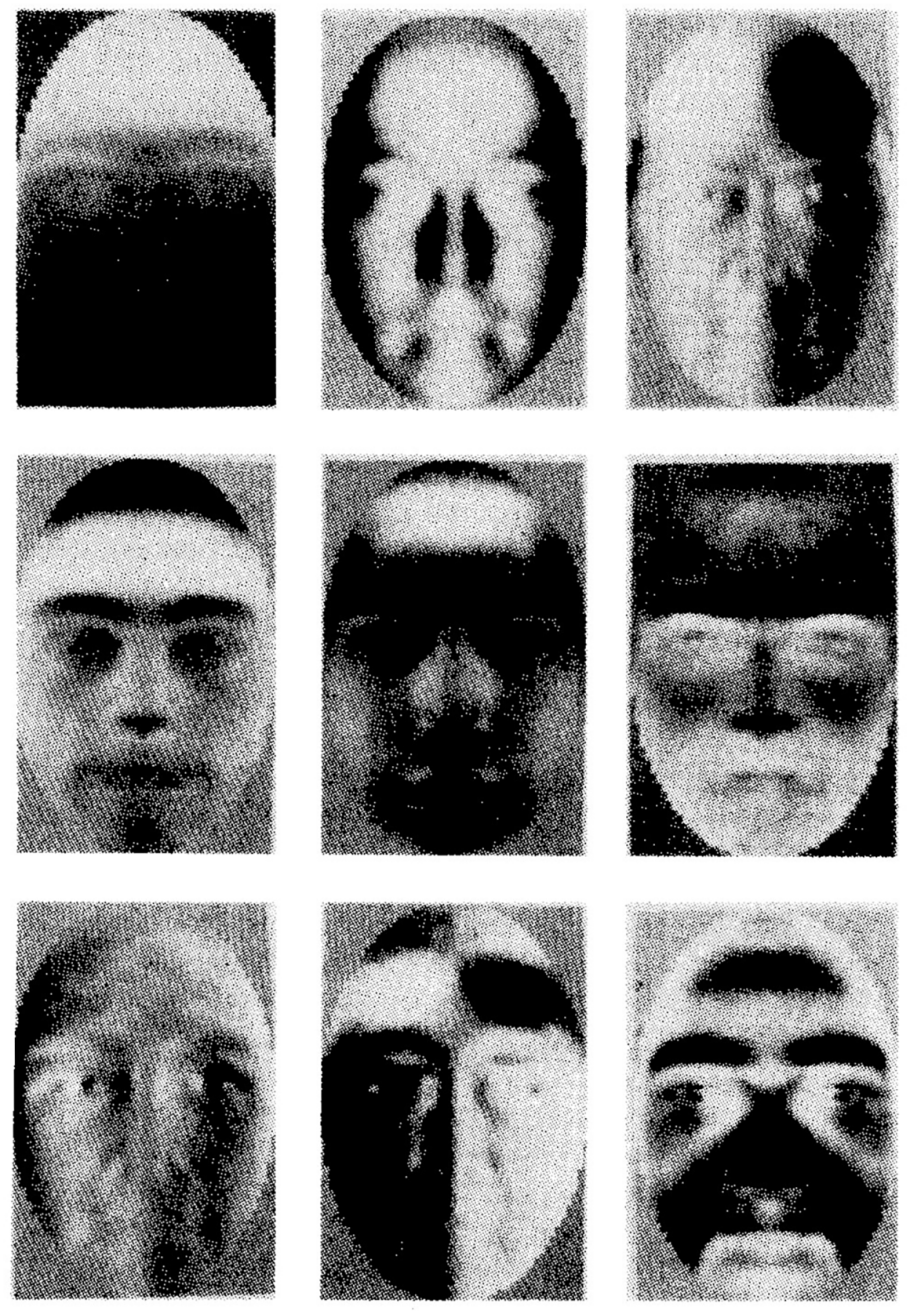
Eigenpictures obtained from a database of 200 Caucasian young male students from Brown University (Kirby & Sirovich, 1990, p. 106).

To produce eigenfaces Turk and Pentland used a database of 200 images from Caucasian males (without beards or eyeglasses) from Brown University (Kirby & Sirovich, 1990, p. 105; Turk & Pentland, 1991, p. 75). From this database, PCA generates ‘eigenpictures’, images that contain the minimal number of features or characteristics that best represent the group of initial pictures (see Figure 4).

Eigenfaces has the advantage of detecting and extracting variation from a group of faces in an automatic way, without the intervention of the researcher and ‘independent of any judgement’ (Turk & Pentland, 1991, p. 73). This means that the analysis of the faces is based on the information found in the pictures (i.e. pixels), rather than on facial features such as eye shape, nose, or lip width. Generated like this, as can be seen in Figure 4, the resulting eigenpictures do not correspond to those that a human observer would select. In this way, the technology promises to take us beyond traditional ways of describing faces, where certain facial traits are racialized, and beyond human stereotypes and biases. However, as I will show, this automatic process enacts race in a different way.

### Race as a physical attribute

To uncover how race is relevant for the eigenfaces algorithm, it is important to understand how facial recognition works. Dr. Roberton explains it as follows: ‘It is a model that has a bunch of templates [i.e. eigenfaces] and you find the best fit between the signal of the image and some combination of the templates. And how much of each template you need is the description’. In other words, recognition works by comparison, by projecting onto the face space new images created by the set of eigenpictures resulting from the database. It is important to understand that eigenpictures are composites created through PCA. They *describe* all the diversity in the database and *prescribe* what a face is. If, for instance, the image projected is too different from the templates and, as a consequence, the eigenpictures found in the face space, the image is not recognized as a face. In the case of the database built with the faces of young, Caucasian, male students from Brown University, faces that are not Caucasian, young and male will probably experience recognition problems. This last case is the most common example of recognition failure reported in more recent algorithms and linked to underrepresentation of specific types of faces in databases (Buolamwini & Gebru, 2018).

In 1991, Kirby and Sirovich described their database as being composed of Caucasian male subjects. What does this mean? ‘Caucasian’, a traditional racial category first introduced by Blumenbach, is considered by Kirby and Sirovich to be a neutral ordering device with a straightforward meaning. In other words, Caucasian is considered an unproblematic fact. This decision has two consequences. On the one hand, labeling this group of faces ‘Caucasian’ makes it possible to name and classify any other group of faces differing from this one using other racial categories, like non-Caucasian, Negroid, Mongoloid, Black American, and Latino, for example. In this way, Kirby and Sirovich define the *terms of description* of normal Caucasian faces. On the other hand, the algorithm has a *prescriptive effect*, in the sense that it determines what counts and what does not count as a Caucasian face. This means that recognized people can be considered ‘Caucasian’, while those who are unrecognized are not only non-Caucasians but are not even recognized as having a face. In this way, race has a prescriptive role through which the previously established set of eigenpictures automatically ‘makes up people’.

The original articles on eigenpictures do not provide details on why this group of Caucasian students was chosen. However, this selection probably mirrors the assumption of homogeneity (which is required to hold for PCA to work). Then, the assumed resemblance is corroborated by PCA. It is taken as intrinsic to the group of photographs and, in turn, as natural to the students that appear in them. Through this process – first selecting similarity, and second automatically detecting and recognizing similarity – racial distinctions become naturalized. Race differences are established as physical attributes that result from the combination of bodies and technologies. This, in turn, strengthens the descriptive and prescriptive role of race and legitimizes the use of this category in facial recognition.

However, PCA is not acting alone, and the attributes described by it are co-produced by the capacities of the cameras that are used. A closer look at this relation (between bodies, cameras, and statistics) can show us how physical attributes and their racial narratives emerge.

### Physical attributes and technology

The criteria underlying the selection of ‘Caucasians’ to assemble training databases are often presented as concerning only technical issues like avoiding low-contrast images and obstacles that obstruct recognition (like glasses). However, these decisions not only influence algorithm performance, but also create a specific *ontology of the normal*. They create a ‘face space’ in which Caucasian university students became the organizing norm.

According to Dr. Roberton, during the early 90s, when eigenfaces was developed, databases were built mostly with white faces for two related reasons: the cameras used and the capabilities of facial recognition. Concerning the first, Dr. Roberton explains: ‘There was a choice back in the ‘70s about what sort of cameras do we use; charged couple devices (CCD) were cheaper and more reliable as opposed to other ones’. However, this ‘fundamental choice of a sensing technology means that black faces have a harder time [being recognized by the camera] than white faces’. According to this argument, the selection of only white faces to train facial recognition algorithms avoids the problems darker faces present for these cameras. In addition, there was a second reason for this selection. Dr. Roberton explains that at that time the main question facing scientists working on facial recognition technologies was whether this technology ‘was real’, whether it could work at all: ‘does it ever work for anyone?’ Interestingly, ‘anyone’ was translated as a white face, which became the default face. Perhaps Dr. Roberton and his colleagues would not have chosen CCD sensing technologies if they had considered it important to recognize non-white faces – if the default face was not considered to be white.

These early decisions have consequences for today’s facial recognition systems. The work on eigenfaces not only implemented racial classifications as a standard approach in facial recognition, it also set the white face as a legitimate default – something the field still suffers from today, in large part. As a result, facial recognition algorithms can be seen as performative technologies that enact and describe race based on physical attributes.

The eigenfaces approach became highly influential (and controversial) in the field (Stanley & Steinhardt, 2002) and went on to have many technological applications. After only a short period of time, the system was used widely for surveillance in casinos, recreational areas and ATMs (Lockie, 2002). It was also used in the first commercial facial recognition application marketed by the company Viisage and used during the Superbowl XXXV in Tampa Bay 2001.

The PCA approach continues to be useful in the production of identification technologies (see Claes et al., 2014). One of these is EvoFIT, software for composite sketching created in 2001 and in use since then in the United Kingdom (Frowd et al., 2019).

## EvoFIT: Evolving composite sketching

In composite sketching, a portrait is produced from the memories of a witness for the purpose of identification (Davies & Valentin, 2006; Mancusi, 2010). In the late 19th century, Alphonse Bertillon (1853–1914), famous for his work in criminal identification, introduced a standardized set of instructions for producing spoken portraits (Bertillon, 1896). After Bertillon, other systems were developed to optimize recognition by making sketches ‘more realistic’. One strategy was to produce photographic catalogs of the most frequently found and expected facial traits (eyes, mouths, chins, noses, face shapes) in a population, that could be combined to produce all possible faces. Two famous systems are Identikit, introduced in the Los Angeles Police department in 1959 (Penry, 1971), and Photo-Fit, introduced in the UK police in 1970 (Higgs, 2011). In both systems, the facial traits included in the photographic set depend on the type of population to be represented. Several updates have been made to the original photographic sets over time. In the case of Photo-Fit, for instance, later versions of the catalog (the 1978 update) included facial traits from male and female Caucasian faces as well as male Afro-Asian faces (Ellis et al., 1978, p. 297).

In recent years, PCA has inspired the creation of EvoFIT, a new composite sketching system that is used to identify criminal suspects (Frowd et al., 2004, 2012; Gibson et al., 2003). One of the two systems used today by police departments in the UK, EvoFIT was produced by Peter Hancock (a computer scientist and a lecturer in psychology at the University of Stirling) and expanded by Charlie D. Frowd (professor of forensic psychology at the University of Central Lancashire). It aims to produce facial composites not by fragmenting a face into facial traits that need to be put together (as Identikit and Photo-Fit do), but rather by using an evolutionary algorithm to focus on whole faces that ‘evolve’. One of the reasons for this change in focus is that, according to Dr. Ricks, people remember faces in a holistic way, rather than trait by trait: ‘You can do something like changing the eyes, and yes it is different, (but) what did you change? You cannot necessarily see it, it is just the whole appearance of the face that changes. And so, it can be quite hard to say what is wrong with the face or what you need to make, it just doesn’t look quite right’. Precisely for this reason, with EvoFIT researchers focus on generating faces that resemble as much as possible the face remembered by a witness, not by asking for descriptions, but by asking for resemblance. Using PCA, the system makes faces ‘evolve’ and allows researchers and police investigators to adjust holistic characteristics, such as masculinity, threat, attractiveness, honesty and extroversion in order to generate faces (EvoFIT, 2020; Frowd et al., 2011). EvoFIT creates families of faces that resemble each other by using eigenfaces for the selected relevant traits. As in the previous case studies, each of these eigenfaces ‘captures the major modes of variation within the image set’ (Frowd et al., 2004, p. 20).

### Race as genealogy

**Figure 5.**
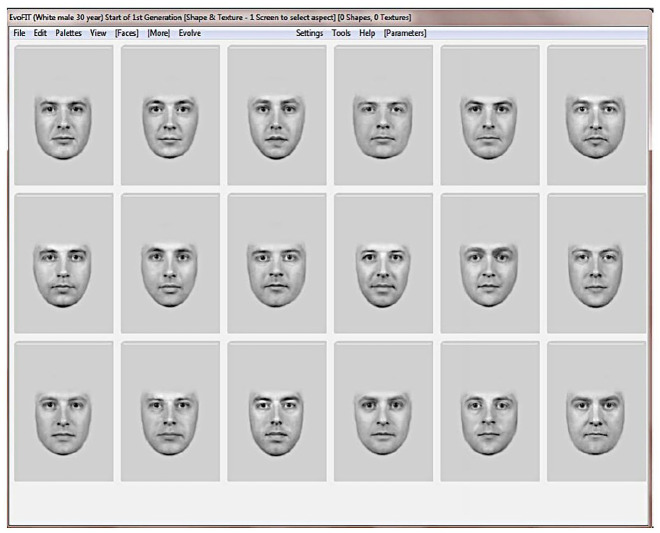
An example of the 18 faces created with EvoFIT shown to a witness. In this case, the faces are generated from a ‘white male 30 years old’ database (Frowd et al., 2012, p. 21).

In a criminal investigation, an investigator interviews the witness and presents exemplars of faces generated using EvoFIT. The witness selects the faces that most resemble the face in her memory. From these selected faces, an evolutionary algorithm produces new faces that in principle are closer to the face in the memory of the witness. Let us say that the witness remembers a young white male. In this case, the investigator presents to the witness 18 faces generated from one specific database, the ‘white male 30 years old’ database (Figure 5). From this selection, the researcher produces new faces. After the witness selects the faces that best represent her memories, the parameters underlying the selected faces are used by the algorithm to produce more faces that resemble those selected. The authors describe this process as a ‘breeding process’ that produces guided variation (Frowd et al., 2012, p. 21). From the selected faces, the one that most resembles the suspect is granted more opportunities *to breed* and the procedure is repeated. In each generation, the families of faces are increasingly homogenous (i.e. faces increasingly resemble each other) and ideally resemble more and more the suspect seen by the witness.

In contrast to the previous case studies, here PCA has a generative role. PCA generates a face space or set of faces in relation to the characteristics of one selected face. In other words, the ontology of the normal set by the ‘parent faces’ directs the breeding process to generate more faces. As a consequence, the resulting faces belong to one single lineage. This implies that to create, for instance, ‘Black’ faces, it is necessary to have ‘Black databases’ and so on. This reproduction of family resemblance evokes racial narratives of genealogy and purity, in which radical phenotypical difference is understood as foreign and admixture as the combination of pure lineages (Gobineau, 1915). As Dr. Ricks explains: ‘[for] a mixed-race person, whatever that means … you might try having a database that consists of some Black guys and some White guys if that is the mixture you are talking about and then the system will produce people intermediate within that space’.

Accordingly, researchers working with EvoFIT in the UK have developed 60 different databases in order to be able to produce ‘different offenders’ (Frowd et al., 2012, p. 25). To produce other families (of faces), it was necessary to ‘breed’ databases containing faces from other populations. Currently, there exist databases for ‘male and female of different ages and races – White, Black, Asian, Eastern European, Chinese, Hispanic and various mixed-race combinations’ (EvoFIT, 2020; Frowd et al., 2012, p. 25). These new populations are then used to breed new lineages or families of faces. Dr. Ricks explains that race is a problematic concept, but that he does not have any other word to refer to the statistical regularities in appearance that can be observed in a population. To him, these statistical regularities are what race stand for. In this regard, the success of PCA in EvoFIT, and generally in facial recognition, is based on the fact that it can extract these regularities in ways believed to be similar to human perception and recognition. In fact, for experts in human recognition, such as Dr. Ricks and Dr. Allison, PCA is more than a good description of how we perceive: It can be said that we are naturally extracting principal components from the faces we look at, that this is simply how *we* work.

Besides this genealogical aspect, there is a second way in which race becomes relevant in EvoFIT. To select which database should be used, the investigator needs to ask the witness about the race, sex, and age of the suspect. In this situation, an agreement between researchers, forensic practitioners, and witnesses is made about the relevance and meaning of these traits (see, Nieves Delgado, 2020b). Thus, race becomes a working category in algorithm-based forensics. It is introduced through a grouping process that is convenient for the purposes of personal identification, i.e. of offender groups. This purpose affects the relationship between user and technology, as well as the creation of relevant data. In this way, technologies not only participate in a context of use but contribute to shape it (Miller, 2021).

## Conclusion

PCA is a statistical method that makes it possible to manage large amounts of data. PCA is widely used in facial recognition because it is believed to resemble how human cognition works. PCA connects our intuitions regarding facial normality with statistical regularities found in datasets. Against this background, I have shown that in facial recognition theories and technologies PCA organizes data according to an *ontology of the normal*. This ontology influences how race is enacted in PCA-based recognition processes: as a type, as a physical attribute, and as a genealogy.

In the first case, researchers produce a database or a ‘face space’ based on their understanding of facial resemblance (and difference) between human groups. It is assumed that, for instance, Caucasians resemble Caucasians and Black people resemble Black people. This assumption is not only highly idealized but also essentially typological. It assumes that there is an intrinsic difference between these groups that does not need to be empirically investigated. Related to this first assumption is the view of race as a set of physical attributes. Like the previous case, it holds that groups labeled as Caucasian or Black are homogeneous sets. However, here the physical attributes of each group empirically redefine racial categories. There is a feedback loop between what researchers assume to be a homogenous set (i.e. races) and the characteristics extracted from these sets though PCA. Consequently, racial labels and categories are once again ‘supported’ by empirical evidence. Lastly, the view of race as genealogy highlights the assumption that each human group shares a common origin. From such lineages result the existence of pure (or admixed) races. The two assumptions of race as physical attribute and as genealogy taken together should explain why people living in one place (country, continent, etc.) resemble each other.

As these case studies show, race works as a central guiding ideal for researchers in their selection of images and human groups. In addition, these cases reveal the recursive nature of race. Race organizes our intuitions on human difference, which are later corroborated by our experiences, experiments and statistics. In this methodological feedback loop, we also find a historical loop: Previous ideas of human sameness are still translated into racial categories that function as guidelines on how to shape experiments and databases. This analysis suggests, more generally, that race in scientific and technological contexts is highly flexible. In fact, race seems to be so flexible that it could qualify as an empty concept, as it is constantly filled with new meanings through new technologies and practices.

It is important to note, however, that the interpretation of statistical groupings through PCA and similar tools does not necessarily require a racial interpretation. In fact, researchers are usually aware of how problematic the use of race is, as, for example, Dr. Ricks expressed during an interview (see also Nieves Delgado, 2020c). What these cases show is that we cannot commit to a simplified and unrealistic generalization of technologies as neutral. Rather, one should see these systems as opening up a possibility space of racialization for researchers and engineers through certain methodological necessities (like homogeneity) and grouping practices (like types or lineages), which trigger stereotypical reasoning.

In light of these findings, we need to take a closer look at how the rapid dissemination of facial recognition algorithms contributes to a renewed use of racial categories. This racialization process has also influenced those working to remove biases in algorithmic recognition (Buolamwini & Gebru, 2018). In fact, recognition biases are commonly explained in terms of racial diversity – or the lack of it in databases. In other words, race is increasingly naturalized by both developers and critics of these technologies. Against this background, this paper has highlighted the different ways in which race is mobilized in facial identification technologies, and the implications of using this category. In contrast to racial science from the past, and the production of portraits, measurements, averages and types, current racialization practices in recognition are immaterial and invisible. However, this invisibility should not lead us to think that these recognition technologies do not have a deep impact on society. Nothing could be further from the truth. In fact, these technologies and their invisible racialization processes are increasingly integrated into policing and surveillance activities, and have various uneven effects on different human groups, sparing privileged groups while criminalizing unprivileged ones. For this reason, we need to inculcate a stronger reflective stance among researchers and developers in facial recognition to further disentangle the role race plays in their work, and how this impacts society.
